# Insights into modeling refractive index of ionic liquids using chemical structure-based machine learning methods

**DOI:** 10.1038/s41598-023-39079-5

**Published:** 2023-07-24

**Authors:** Ali Esmaeili, Hesamedin Hekmatmehr, Saeid Atashrouz, Seyed Ali Madani, Maryam Pourmahdi, Dragutin Nedeljkovic, Abdolhossein Hemmati-Sarapardeh, Ahmad Mohaddespour

**Affiliations:** 1 Renewable Energies Engineering Department, Faculty of Mechanical and Energy Engineering, Shahid Beheshti University, Tehran, Iran; 2 Department of Chemical Engineering, Amirkabir University of Technology (Tehran Polytechnic), Tehran, Iran; 3 Department of Chemical and Petroleum Engineering, University of Calgary, 2500 University Drive NW, Calgary, AB T2N 1N4 Canada; 4 Department of Polymer Reaction Engineering, Faculty of Chemical Engineering, Tarbiat Modares University, Tehran, Iran; 5 College of Engineering and Technology, American University of the Middle East, Egaila, 54200 Kuwait; 6 Department of Petroleum Engineering, Shahid Bahonar University of Kerman, Kerman, Iran; 7 State Key Laboratory of Continental Shale Hydrocarbon Accumulation and Efficient Development, Ministry of Education, Northeast Petroleum University, Daqing, 163318 China; 8 Department of Chemical Engineering, McGill University, Montreal, QC H3A 0C5 Canada

**Keywords:** Chemistry, Engineering, Computer science

## Abstract

Ionic liquids (ILs) have drawn much attention due to their extensive applications and environment-friendly nature. Refractive index prediction is valuable for ILs quality control and property characterization. This paper aims to predict refractive indices of pure ILs and identify factors influencing refractive index changes. Six chemical structure-based machine learning models called eXtreme Gradient Boosting (XGBoost), Light Gradient Boosting Machine (LightGBM), Categorical Boosting (CatBoost), Convolutional Neural Network (CNN), Adaptive Boosting-Decision Tree (Ada-DT), and Adaptive Boosting-Support Vector Machine (Ada-SVM) were developed to achieve this goal. An enormous dataset containing 6098 data points of 483 different ILs was exploited to train the machine learning models. Each data point’s chemical substructures, temperature, and wavelength were considered for the models’ inputs. Including wavelength as input is unprecedented among predictions done by machine learning methods. The results show that the best model was CatBoost, followed by XGBoost, LightGBM, Ada-DT, CNN, and Ada-SVM. The R^2^ and average absolute percent relative error (AAPRE) of the best model were 0.9973 and 0.0545, respectively. Comparing this study’s models with the literature shows two advantages regarding the dataset’s abundance and prediction accuracy. This study also reveals that the presence of the –F substructure in an ionic liquid has the most influence on its refractive index among all inputs. It was also found that the refractive index of imidazolium-based ILs increases with increasing alkyl chain length. In conclusion, chemical structure-based machine learning methods provide promising insights into predicting the refractive index of ILs in terms of accuracy and comprehensiveness.

## Introduction

By definition, ionic liquids (ILs) are molten salts with melting points of less than 100 °C; thus, they remain liquid about and under room temperature. Low volatility and vapor pressure, satisfactory chemical stability, high conductivity, and being suitable solvents have given ILs several applications in various fields^1^. Photovoltaic cells^2^, batteries^3^, thermo-electrochemical cells^4^, water treatment technologies^5^, thermal energy storage devices^6^, carbon capture^7^, and other green utilizations^8^ are among these applications. In order to analyze the purity of an IL or to obtain useful information about the behavior of molecules in a solution, an optical property called refractive index is investigated^9^. IUPAC has defined refractive index as “the ratio of the speed of light in vacuum to that in a given medium”^9^, and it has recently gained attention in the quality control and characterization of ILs subject^10^. Because acquiring the physical properties of ILs through experiments is not efficient in terms of time and cost^11^, in recent years, many researchers have been inclined to develop general models that can predict the physical properties of diverse ILs^12^. In addition, It is not realistic to experimentally screen ILs with preferable properties considering that the number of potential ILs is predicted to be 10^8^^13^. Predicting these physical and thermodynamic properties of ILs can be performed using machine learning methods, which are increasingly advancing in research fields^11^. It has been a while since researchers began to investigate applying machine learning methods to predict different properties of ILs^14^, such as viscosity^15^, electrical conductivity^16^, thermal conductivity^17^, gas solubility^18^, and surface tension^19^. The fantastic improvement of machine learning has shown a new perspective to scientists^12^.

Many models have been proposed and used in the literature to predict the refractive index of ILs. Iglesias-Otero et al.^20^ suggested a correlation to determine the density of a binary IL, which can also be used to predict the refractive index through inverse prediction. The differences between the correlation and 131 experimental data points were less than 0.001. Another correlation was used by Koller et al.^21^ for 32 data points, and an average RMS of 0.007 was reported. Likewise, Safdar et al.^22^ suggested a correlation equation using 35 data points, leading to an R^2^ > 0.99 and an average standard deviation of 0.023. In addition, Tong et al.^23^ used a semiempirical method with 25 data points with an R^2^ of 0.99 and an average standard deviation of 2.78 × 10^–5^. Similarly, Xu et al.^24^ presented a semiempirical method using 35 data points resulting in an R^2^ of 0.99 and an average standard deviation of 4.15 × 10^–5^. Other researchers used a group contribution method to estimate the refractive index of ILs. Gardas et al.^25^ gathered 245 data points from literature and obtained an AARD of 0.18% using a group contribution method. Almeida et al.^26^ also used a group contribution method utilizing 105 data points and found an AARD of 0.02%. Sattari et al.^27^ brought together a more extensive database of 931 data points and found the AARD of 0.34%, R^2^ = 0.964, and RMSE of 9.97 × 10^–3^ using a group contribution method.

Researchers also included machine learning methods in their predictions. Díaz-Rodríguez et al.^28^ put their effort into using artificial neural networks in the form of multilayer perceptrons (MLP) and compared their accuracy with multiple linear regression (MLR) models. After analyzing 72 data points and reporting R^2^ and mean prediction error (MPE) of both MLP and MLR models, being R^2^_MLP_ = 0.98, R^2^_MLR_ = 0.76; MPE_MLP_ = 0.24%, MPE_MLR_ = 0.72%, they concluded that MLP method was quite convincing in predicting the refractive indices of pure ILs, while MLR model was not accurate enough for their system. In another research, Díaz-Rodríguez et al.^29^ used the MLP method with a data size of 156 to predict the refractive index of ILs. They found the R^2^ to be more than 0.99 and MPE of 0.02%, which were satisfactory. Furthermore, Díaz-Rodríguez et al.^30^ employed two MLP models and predicted the refractive index of different pure ILs with MPE of less than 0.48% using 39 data points. In another study, Golzar et al.^31^ used 85 data points for predicting the refractive index of ILs by conducting the ANN method. The result showed that R^2^ was very close to unity. Similarly, Cancilla et al.^32^ used 72 data points of ternary ILs to develop their ANN model and estimated the refractive indices with an MPE of 0.05%. In their next paper, Cancilla et al.^33^ expanded their dataset to 146 and utilized four models based on MLPs to estimate the refractive index of ILs with MPE of less than 1%. Recently, researchers generally tend to expand their databases. Soriano et al.^34^ made use of a database including 752 data points of binary ILs in their ANN model with a mean absolute error of 0.00783 and an overall average percentage error of 0.55%. Mesbah et al.^12^ used 362 data points to model ternary systems through separate models: ANN and GEP. Their ANN model results had an R^2^ of 0.9225, MSE of 2.47 × 10^–5^, and AARD% of 0.2773 in predicting refractive index, while the error analysis of the refractive index correlation provided by GEP showed an R^2^ of 0.9765, MSE of 7.20 × 10^–6^ and AARD% of 0.1383. Kang et al.^35^ also used a machine learning method called ELM to model a number of 1194 data points and compared the results with those obtained from the MLR method. The R^2^ and AARD% values obtained by MLR were 0.841 and 0.855%, respectively whereas they were 0.957 and 0.295%, respectively for the ELM model. They concluded that ELM method was better than MLR in predicting the refractive indices. Another large database consisting of 3147 data points were used by Venkatraman et al.^10^ to model a refractive index of ILs by various machine learning models. They presented an MAE of less than 0.01 and an R^2^ of more than 0.85 across both test and training data. A different study by Soroush et al.^11^ was done using an ANN model with 812 data points and has an R^2^ of 0.9993, an MSE of 6.91 × 10^−7^, and an AARD% of 0.04 for estimating the refractive index of various ternary ILs. An MLP model was used by Wang et al.^36^ with 688 data points of different binary systems and achieved average error parameters of MSE = 8.45 × 10^–6^, R^2^ = 0.9905, AARD = 2.42%.

Sattari et al.^9^ proposed a QSPR model for estimating the refractive index of ILs using 931 data points. Their statistical analysis showed an R^2^ of 0.935, RMSE of 1.07 × 10^−2^, and ARRD of 0.51%. Other researchers tried combining machine learning methods with different models to obtain better results. Wang et al.^37^ performed and compared a Group Contribution-Artificial Neural Network (GC-ANN) model with a Group Contribution model utilizing 2138 data points. Providing that the AARDs of the GC-ANN model and GC model were 0.179% and 0.628%, respectively; while the R^2^ of the GC-ANN model and GC model were 0.961 and 0.886 respectively, they concluded that the ANN-GC model exhibited a better outcome. In another study, Ding et al.^38^ gathered two datasets of refractive indices consisting of 3147 (first dataset) and 931 (second dataset) data points to build an XGBoost-assisted QSAR model capable of predicting the refractive index of a various number of ILs. They used two molecular fingerprints (MF) named Morgan fingerprint and atom-pair fingerprint as two molecular descriptors. The outcome error of their model was RMSE = 0.017 and R^2^ = 0.782 for the first dataset using the Morgan fingerprint descriptor, and RMSE = 0.013 and R^2^ = 0.853 for the second dataset. Also, an RMSE of 0.016 and R^2^ of 0.836 was obtained for the first dataset utilizing atom-pair fingerprint, and RMSE of 0.022 and R^2^ of 0.568 for the second dataset. Furthermore, Sun et al.^13^ used 3964 data points and four ways of IL descriptors to develop their QSPR-XGBoost model and assess its predictive performance. They concluded that the QSPR model developed by molecular fingerprint combined with molecular descriptor has the best accuracy with R^2^ of 0.951 and RMSE of 0.0088. Recently, Baskin et al.^39^ predicted the refractive index of various ILs using a collected 6443 data points. They utilized several machine learning methods to develop their QSPR models using different molecular descriptors. Once they predicted the refractive indices in one temperature, then they performed their estimation varying 8 different temperatures. They concluded that the best ML method for one-temperature and eight-temperature investigations were ASNN and DNN, respectively. Additionally, the best representation for their QSPR model to predict the refractive index were CDK23 in both investigation cases. The statistical outcome of their research was an R^2^ of 0.86, an RMSE of 0.016, an MAE of 0.0081 for only one temperature, and an R^2^ of 0.922, an RMSE of 0.0112, and an MAE of 0.00725 for when they changed the temperature in the specified range. As the literature review illustrated, the studies that used methods other than machine learning incorporated few ILs and data points for modeling, whereas the studies that developed machine learning with many data points obtained insignificant accuracy. Further, using wavelength as an input in machine learning models has not yet been investigated.

This study uses six robust chemical structure-based machine learning models named XGBoost, LightGBM, CatBoost, CNN, Ada-DT, and Ada-SVM to predict the refractive indices of an extensive database, including the temperature, wavelength, and chemical substructures of each data point as inputs. The database contains 6098 data points from 483 ILs. Besides, this study investigates how each chemical substructure, temperature, and wavelength affect the refractive index. The best model is also introduced through statistical analysis and graphical representation.

### Data collection

The dataset used in this study was obtained from the NIST Ionic Liquids Database (SRD#147 v2.0)^40,41^. The extracted data comprise 6098 data points belonging to 483 different pure ILs. The temperatures under which these ILs were tested varied from 278.15 to 368.1 K. The wavelength in the experimental data changed from 430.1 to 822.7 nm, resulting in refractive index changes of 1.335–1.7. Additionally, the molecular weights of the ILs used in this study differ from 77.08 to 866.64 g/mol. Brief statistics of the data used in this study are presented in Table 1.

**Table 1:** Statistical characteristics of the gathered data.

Parameter	Symbol	Unit	Mean	Min	Max
Molecular weight	Mw	g/mol	318.62	77.08	866.64
Temperature	T	K	313.39	278.15	368.1
Wavelength	$\uplambda$	nm	590.69	430.1	822.7
Experimental refractive index	n_D_	–	1.460	1.335	1.700

Table S1 in Supplementary Information section presents all the ILs used in the present study with the ranges of their temperature, wavelength, and refractive index, as well as the number of corresponding data points.

**Figure 1:**
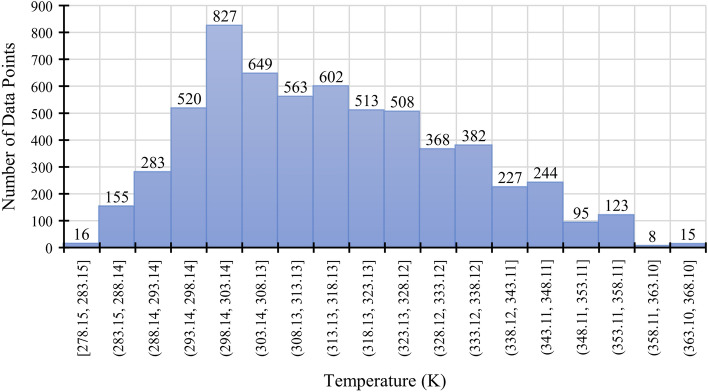
Dispersion of input data points over the temperature range.

**Figure 2:**
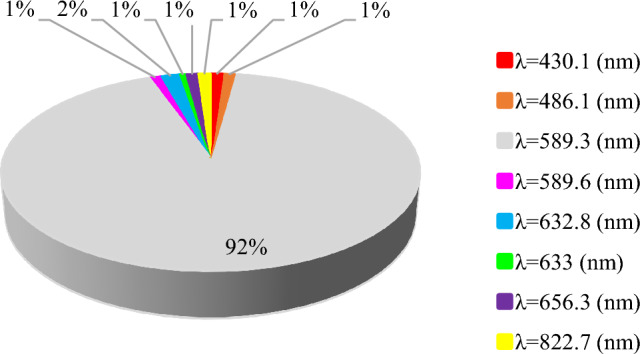
Wavelength allocation of the input data.

Graphical demonstrations showing the dispersion of the database regarding the temperature and wavelength are presented in Figs. 1 and 2. Figure 1 shows that most data points lie between 298.14 K and 303.14 K. Moreover, as shown in Fig. 2, the majority of the wavelengths have a wavelength of 589.3 nm, which is the sodium D line wavelength. The scarcity of the other wavelengths should not be interpreted such that this parameter has a negligible role in determining the refractive index. As the literature states, the pressure, temperature, composition, and the light source wavelength are the variables that correlate with the refractive indices of liquid mixtures^42^. Cauchy’s equation and the Sellmeier equation used by Guo et al.^43^ and Arosa et al.^1^, respectively serve the purpose of showing the empirical relation between wavelength and the refractive index. Thus, the influence of wavelength on the refractive index of ionic liquids is taken for granted by the literature.

**Figure 3:**
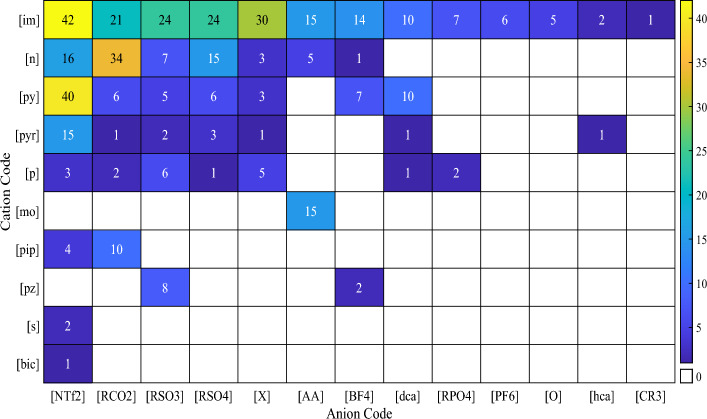
The number of the used ILs regarding their cation and anion families combinations.

Each ionic liquid consists of anionic and cationic families. These family names and the number of ILs containing them are accumulated in Table 2. The cationic and anionic family combinations used in the current study with the number of ILs comprising them are displayed in Fig. 3. It can be realized that the most repeated ionic liquid family in the database is [im][NTf2].

**Table 2:** Cation and anion family names, their code, and their number used in this paper.

Family name	Code	Number of distinct ions
Cations		
Ammonium	[n]	85
Imidazolium	[im]	225
Phosphonium	[p]	20
Pyridinium	[py]	78
Sulfonium	[s]	2
Piperidinium	[pip]	14
Pyrrolidinium	[pyr]	24
Morpholinium	[mo]	15
Pyrazolium	[pz]	10
Bicyclic	[bic]	1
Anions		
Carboxylates	[RCO_2_]	77
BF_4_ derivatives	[BF_4_]	24
Dicyanamides	[dca]	22
Sulfonates	[RSO_3_]	52
Inorganics	[X]	45
PF_6_ derivatives	[PF_6_]	6
NTf_2_ derivatives	[NTf_2_]	124
Phosphates	[RPO_4_]	9
Sulfates	[RSO_4_]	51
Amino acids	[AA]	35
Alcoholates	[O]	5
Methanides	[CR_3_]	1
Heterocyclic amines	[hca]	3

## Modeling

### Modeling procedure

**Figure 4:**
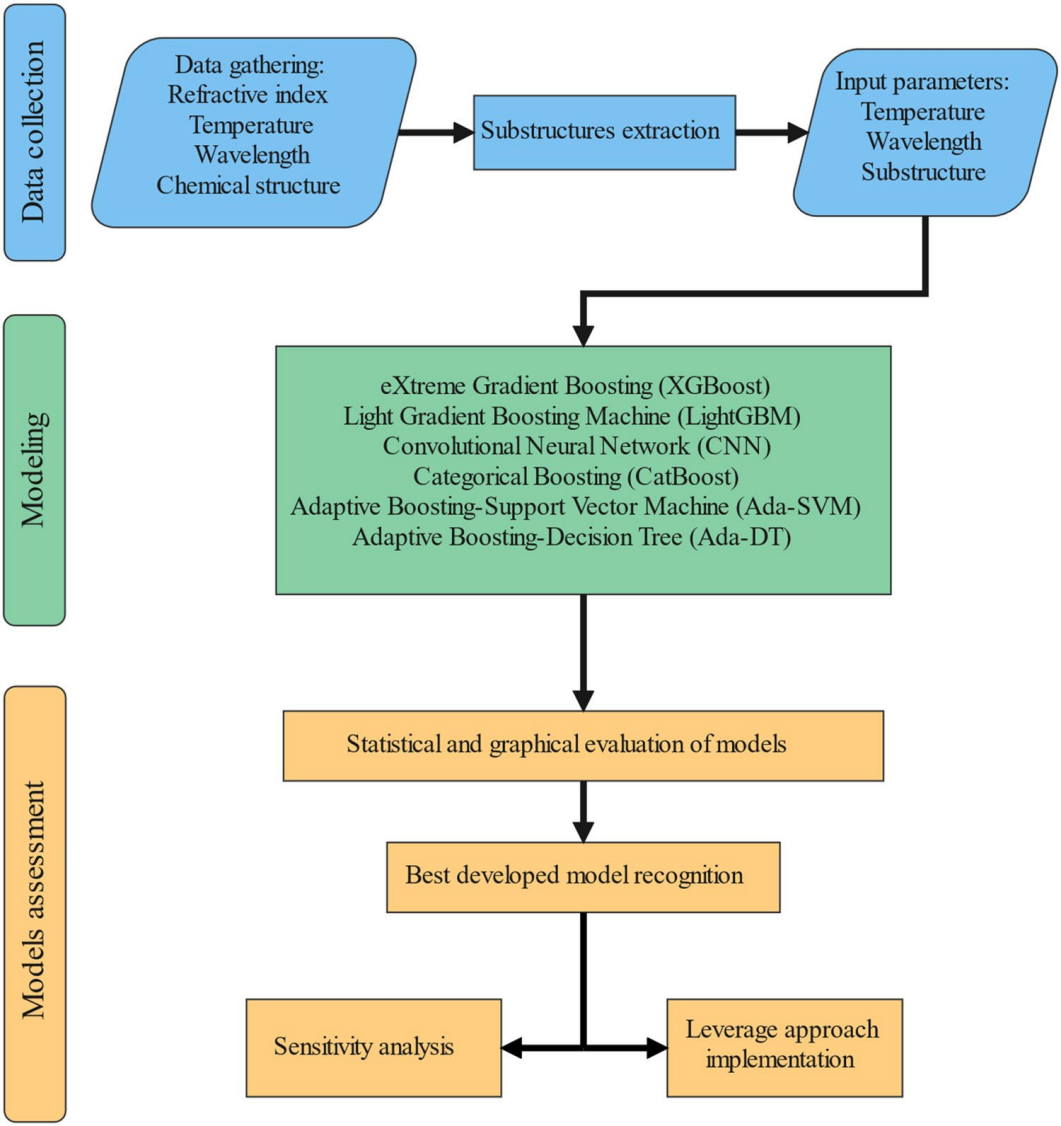
Flowchart of this research’s steps.

The process of modeling, starting with data gathering and ending with results analysis, is provided in the flowchart in Fig. 4. The strong models used in this study are CatBoost, XGBoost, LightGBM, Ada-DT, CNN, and Ada-SVM. The following sections present extensive descriptions of the used models, their hyperparameters, and their inputs.

### Model development

#### XGBoost

XGBoost is a scalable tree boosting system that is one of the most successful and broadly used methods of machine learning. The model choice of XGBoost is called “decision tree ensemble”, which comprises a set of classification and regression trees (CARTs). Because using one single tree is not practically enough, an ensemble model that sums the prediction of various trees is commonly used^44^. The model can be written as Eq. (1):

1 $${\widehat{y}}_{i}=\sum_{k=1}^{K}{f}_{k}\left({x}_{i}\right), {f}_{k}\in \mathcal{F},$$

where $K$ is the number of trees, ${f}_{k}$ is a function in the regression tree space $\mathcal{F}$, and $\mathcal{F}$ is the set of all possible CARTs. To train the model, the objective function is defined and minimized.

2 $$L=\sum_{i}l\left({\widehat{y}}_{i},{y}_{i}\right)+\sum_{k}\Omega \left({f}_{k}\right),$$

where $\Omega \left({f}_{k}\right)=\gamma T+\frac{1}{2}\lambda {\Vert \mathcalligra{w} \Vert }^{2}$. The first term of Eq. (2) indicates the training loss, and the second one is the regularization term which controls the complexity of the model. The regularization term is necessary to avoid overfitting. Since it is intractable to learn all the trees at once, XGBoost uses an additive training strategy. Therefore the prediction of the i-th instance of the t-th iteration is substituted to the objective function.

3 $$L=\sum_{i}l\left({y}_{i},{\widehat{y}}_{i}^{\left(t-1\right)}+{f}_{t}\left({x}_{i}\right)\right)+\Omega \left({f}_{k}\right).$$

By adding a new tree at a time, new predictive values are generated step by step^44^.

4 $${\widehat{y}}_{i}^{\left(t\right)}={\widehat{y}}_{i}^{\left(t-1\right)}+{f}_{t}\left({x}_{i}\right).$$

#### LightGBM

**Figure 5:**
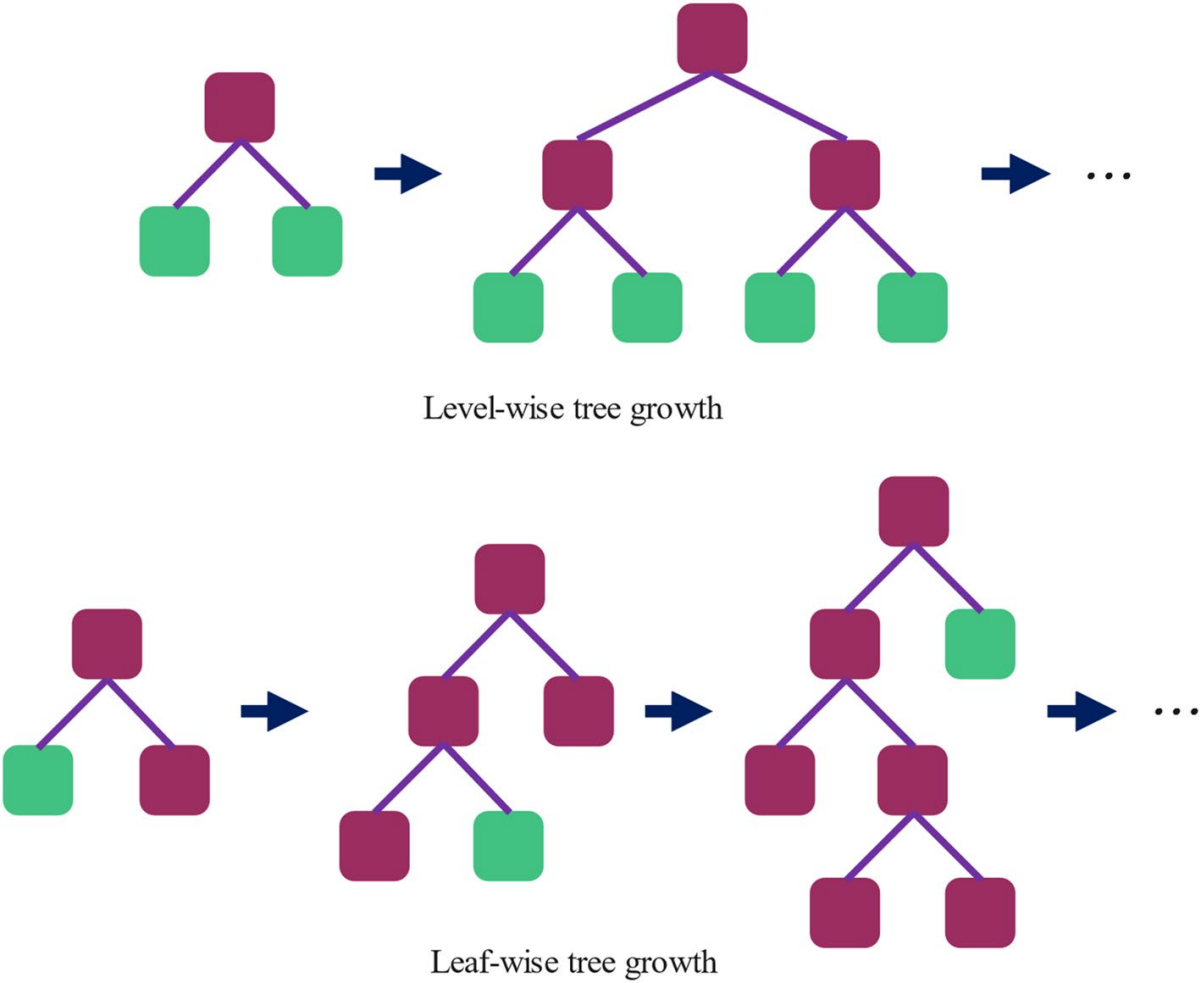
Level-wise and leaf-wise tree growth comparison^46^.

Another algorithm besides XGBoost, which uses the GBDT framework, is LightGBM. The primary purpose of this method is to increase computational efficiency so the predicting problem can be carried out more effortlessly^45^. LightGBM has two features that help solve the problem more cost-effectively: histogram-based decision tree algorithm and leaf-wise growth strategy. Unlike XGBoost and many boosting tools, which use pre-sort-based algorithms for decision tree learning, LightGBM uses histogram-based algorithms. In a histogram-based decision tree algorithm, floating-point eigenvalues are discretized into bins used to construct the histogram. Once the histogram accumulates gradients and samples within each compartment, we can find the optimal segmentation point using the discrete value of the histogram. In accordance with Fig. 5, in a level-wise growth approach, the leaves on each layer are separated at the same time. This strategy is inefficient regarding memory consumption because many leaves have low information gain, and it is unnecessary to search and split them. Leaf-wise growth approach, instead, splits only the leaves with the most significant information gain on each tree layer. This strategy reduces memory usage and speeds up training^45,46^.

#### CatBoost

CatBoost is a gradient boosting method that primarily aims to decrease the prediction shift during data training^47^. The prediction shift arises from a particular type of target leakage present in all implementations of gradient boosting algorithms^48^. One of the benefits of CatBoost is that it uses an innovative algorithm that treats categorical features as numerical characteristics. It also combines category features that exploit the connections between features, enriching feature dimensions. In addition, it employs a symmetrical tree model to overcome the overfitting problem, so the algorithm becomes more accurate and generalized^49^.

#### Convolutional neural network

In the literature, CNN architectures can be found in a wide variety of variations; however, they are all based on similar fundamental principles. A sample CNN comprises three layers other than input and output layers: convolutional layer, pooling layer, and fully-connected layer. Learning characteristic representations of the inputs is the purpose of the convolutional layer. By reducing the feature maps’ resolution, the pooling layer achieves shift-invariance. Some fully-connected layers may exist after several convolutional and pooling layers. The final layer of a CNN is the output layer. Minimizing a loss function on a particular task can obtain the optimal parameters for that task. The CNN loss function is defined as follows^50^:

5 $$L=\frac{1}{N}\sum_{n=1}^{N}\mathcalligra{l}\left({\varvec{\theta}};{{\varvec{y}}}^{\left(n\right)},{{\varvec{o}}}^{(n)}\right),$$

where $N$ is the number of desired input–output connections, ${\varvec{\theta}}$ is all the parameters of the CNN, ${{\varvec{y}}}^{\left(n\right)}$ is the n-th data’s corresponding target label, and ${{\varvec{o}}}^{(n)}$ is the output of the CNN. The best fitting parameters can be obtained by minimizing the $L$ function. As a matter of fact, training a CNN is a global optimization problem^50^.

#### AdaBoost

Generally, boosting is a solid approach for increasing regression and classification models’ predictive power and accuracy. AdaBoost is a boosting algorithm that performs in the 4 following steps^51^:

1. The input containing the number of cycles, a learning algorithm, and a set of training samples are given to the AdaBoost algorithm.
2. AdaBoost gives a number of identical weights to all training samples.
3. It calls the algorithm to train a classifier regarding the weighted training samples and calculates the error. Then, it sets the weight for the component classifier and updates the weight of the training samples in some defined loops.
4. This procedure advances the specified cycles, and finally, AdaBoost linearly integrates all the component classifiers into a single output.

#### Decision tree

Decision trees work by splitting a dataset sequentially into small segments until the target variables match or until the dataset cannot be divided anymore. The algorithm is greedy because it makes the best decision at the given time without considering global optimality^52^. There are different types of DT algorithms, but all of them use a similar structure explained in the following steps^53^:

1. Assigning every training instance to the tree’s root and setting the node as the tree’s root node.
2. Finding the split characteristic and value in accordance with the split criterion. The criterion might be the Gini coefficient, information gain, or information gain ratio.
3. Using the split feature and threshold number to divide all data points in each node.
4. Designating all partitions of the current node as child nodes.
5. Tagging every child node as a leaf and returning for child nodes with only one class instance; otherwise, setting the node as the current node and returning to step 2.

#### Support vector machine

**Figure 6:**
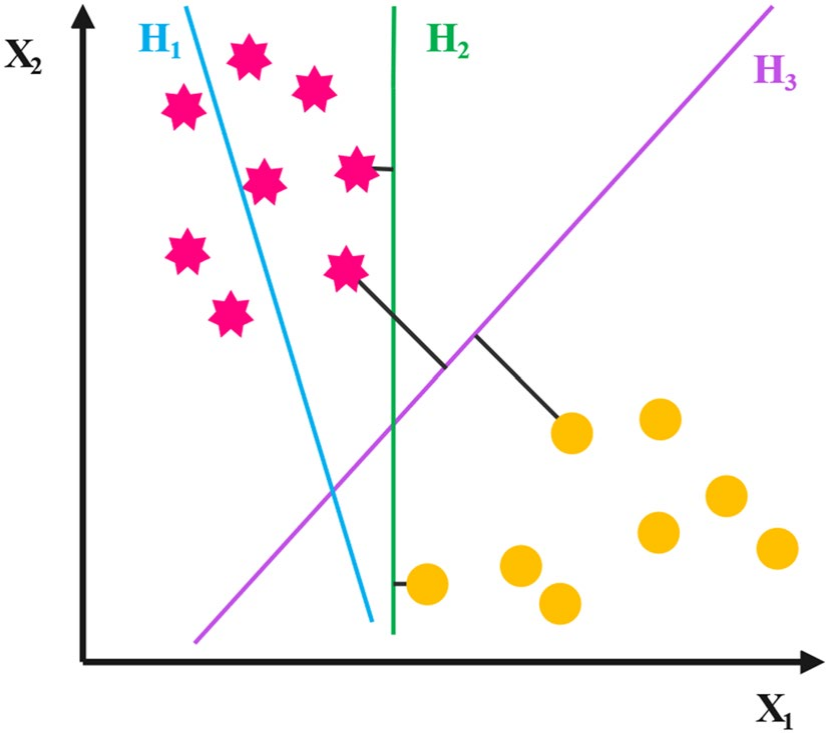
Three hyperplanes separating data: H_1_ that fails to classify correctly, H_2_ with a small margin, and H_3_ with the maximal margin.

Support Vector Machine is another famous supervised learning algorithm that can be utilized for both regression and classification problems. This algorithm plots data points as a single point in an n-dimensional space, where n is the number of inputs. The target of the SVM algorithm is to make the best line to separate the n-dimensional space into discrete classes so that new data points can be put in the appropriate category later. This line is called a hyperplane; the farther it is from the points of any class, the better separation is achieved. Thus, there is not only one hyperplane capable of separating the data, but the best one is the one with the most significant margin between the two classes. It is also worth mentioning that the closest points to the hyperplanes are called support vectors. Figure 6 shows two hyperplanes with small and maximal margins (H_2_ and H_3_) and one that fails to separate the classes correctly(H_1_). The decision function in the SVM algorithm is^51^:

6 $$f\left({\varvec{x}}\right)=\langle {\varvec{w}},\varnothing \left({\varvec{x}}\right)\rangle +b,$$

where $b$ is the bias term, $\varnothing \left({\varvec{x}}\right)$ is a mapping of ${\varvec{x}}$ from the input space to the n-dimensional feature space, and ${\varvec{w}}$ is the weight of the sample. To obtain the optimal values of ${\varvec{w}}$ and $b$, the following optimization problem has to be solved:

7 $$\mathrm{minimize}: g\left(w,\xi \right)=\frac{1}{2}{\Vert w\Vert }^{2}+C\sum_{i=1}^{n}{\xi }_{i},$$

8 $$\mathrm{subject \; to}: {y}_{i}\left(\langle w,\phi \left({x}_{i}\right)\rangle +b\right)\ge 1-{\xi }_{i}, \quad {\xi }_{i}\ge 0,$$

where the regularization parameter is $C$ and ${\xi }_{i}$ is the i-th slack variable^51^.

### Hyperparameters optimization

Adapting a machine learning model to different problems requires tuning its hyperparameters. As a result, choosing the appropriate hyperparameter configuration is a crucial step in the development of machine learning models, as it directly affects their performance^54^. In Table 3, all the adjusted hyperparameters have been provided in addition to their range of analysis.

**Table 3:** Hyperparameters with their corresponding ranges.

Model	Parameters and ranges	Selected model
XGBoost	*n_estimators*: [100–500, step = 50] *learning_rate*: [0.01, 0.02, 0.5, 0.1] *max_depth*: [5–15, step = 1] *subsample*: [0.8, 0.9, 1] *booster*: ['gbtree', 'gblinear', 'dart']	*n_estimators: 100* *learning_rate: 0.1* *max_depth: 10* *subsample: 0.9* *booster:* 'dart'
LightGBM	*learning_rate*: [0.1, 0.2, 0.5, 0.8] *num_leaves*: [5, 10, 20, 30, 40, 50] *max_depth*: [5–15, step = 1]	*learning_rate: 0.2* *num_leaves: 50* *max_depth: 10*
CatBoost	*depth*: [5–15, step = 1], *learning_rate*: [0.01, 0.02, 0.04, 0.08, 0.1] *loss_function*: [‘RMSE, ‘MAE’]	*depth:10* *learning_rate: 0.2* *loss_function: ‘RMSE’*
CNN	(Selected Model)	Conv1D layer parameters: Filters: 32 Kernel size: 2 Activation function: ReLU 1st Dense layer parameters: Number of neurons: 64 Activation function: ReLU 2nd Dense layer parameters: Number of neurons: 1 No activation function (linear layer)
Ada-SVM	*n_estimators*: [100–500, step = 50] *kernel*: ['linear', 'poly', 'sigmoid', 'rbf'] *gamma*: ['scale', 'auto'] *C*: [0.1–1, step = 0.1] *epsilon*: [0.02–0.1, step = 0.02]	*n_estimators:100* *kernel:* 'rbf' *gamma:* 'scale' *C: 1* *epsilon: 0.02*
Ada-DT	*n_estimators*: [100–500, step = 50] *max_depth*: [2–10, step = 1] criterion: ['mse', 'friedman_mse', 'mae'] *splitter*: ['best', 'random'] *max_features*: ['auto', 'sqrt', 'log2']	*n_estimators*: 100 *max_depth*: 9 criterion: 'mae' *splitter*: 'best' *max_features*: 'auto'
Ada-SVM	*n_estimators*: [100–500, step = 50] *kernel*: ['linear', 'poly', 'sigmoid', 'rbf'] *gamma*: ['scale', 'auto'] *C*: [0.1–1, step = 0.1] *epsilon*: [0.02–0.1, step = 0.02]	*n_estimators:100* *kernel:* 'rbf' *gamma:* 'scale' *C: 1* *epsilon: 0.02*

### Input parameters

The independent inputs of our models are temperature, wavelength, and chemical substructure of the ILs. Since the temperature has a well-known influence on the refractive index, it is included in model inputs^37^. However, the wavelength never caught the researcher’s attention to be considered as input to their machine learning models. Numerous studies focused on analyzing and determining the refractive index at a single wavelength called the sodium D line, which equals 589.3 nm. A material’s chromatic dispersion, which is the variation of the refractive index over a range of wavelengths, will be left out of the study if the wavelength is excluded in modeling. Furthermore, much information about the chemical composition and physical properties can be obtained by considering the wavelength as input^1^.

While dispersion is minimized in certain applications (for instance, optical communications systems and imaging systems), it benefits other applications (for instance, dispersive prisms in laser cavities, for compensating dispersion introduced by other optical components, or in optical spectrometers). The refractive index dispersion of an optical device must be accurately characterized in both cases in order to ensure optimal performance^55^.

**Figure 7:**
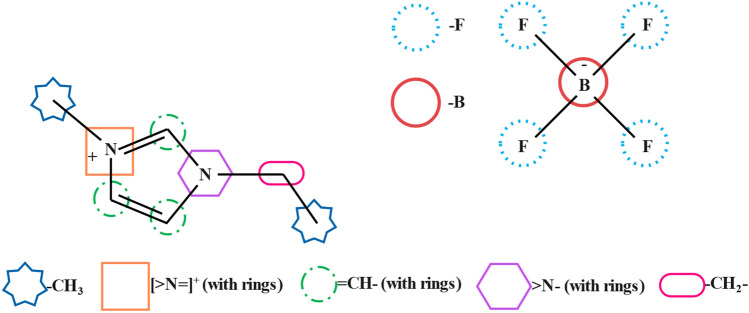
Fragmentation of an ionic liquid into its substructures.

The chemical substructure of an ionic liquid was also used as input in the models, similar to what Valderrama et al.^56^ proposed. A list of the chemical substructures used in this study is presented in Table 4. Figure 7 shows a sample of the process in which an ionic liquid can be fragmented into its substructures. In Fig. 7, the cation of the presented ionic liquid has been fragmented into two –CH_3_, one [> N =]^+^ (with rings), three = CH– (with rings), one > N– (with rings), and one –CH_2_– substructures. Likewise, the anion part of the ionic liquid can be fragmented into four –F and one –B substructures. It is also worth mentioning that pressure was not included in the inputs because the pressure change has little effect on the refractive index^57^.

**Table 4:** A set of 36 chemical substructures utilized in this study.

	Substructures	
Without rings	–CH_3_	–NH_2_
	–CH_2_–	–NH_3_
	>CH–	–NH–
	(>C<) OR ([>C–]^−^)	(>N–) OR ([–N–]^−^)
	=CH_2_	–CN
	=CH–	–NO_2_
	=C<	–F
	–OH	–Cl
	(–O–) OR ([–O]^−^)	–Br
	>C=O	–I
	–COOH	–P
	–COO–	–B
	–HCOO–	–S–
	=O	O=S=O
With rings	–CH_2_–	–O–
	>CH–	–NH–
	=CH–	(>N–) OR ([>N<]^+^)
	=C <	(=N–) OR ([>N=]^+^)

## Assessment of models

### Statistical assessment

It is crucial to evaluate the proposed models’ accuracy by statistical and graphical analysis of the results. Statistical model performance is assessed through the use of a set of equations known as average absolute percent relative error (AAPRE), coefficient of determination (R^2^), root mean square error (RMSE), and mean absolute error (MAE).

9 $$AAPRE=\frac{1}{N}\sum_{i=1}^{N}\left|\frac{{x}_{i,exp}-{x}_{i,pred}}{{x}_{i,exp}}\right|\times 100,$$

10 $${R}^{2}=1-\frac{\sum_{i=1}^{N}{\left({x}_{i,exp}-{x}_{i,pred}\right)}^{2}}{\sum_{i=1}^{N}{\left({\overline{x} }_{exp}-{x}_{i,exp}\right)}^{2}},$$

11 $$RMSE=\sqrt{\frac{1}{N}\sum_{i=1}^{N}{\left({x}_{i,exp}-{x}_{i,pred}\right)}^{2}},$$

12 $$MAE=\frac{\sum_{i=1}^{N}\left|{x}_{i,exp}-{x}_{i,pred}\right|}{N},$$

where $N$ is the number of data points, ${x}_{i,exp}$ is the i-th experimental value of the refractive index, ${x}_{i,pred}$ is the i-th predicted value of the refractive index, and ${\overline{x} }_{exp}$ is the average value of the experimental amount of the refractive index.

### Graphical assessment

In addition to statistical assessment, the model can be evaluated through visual plots containing cross plots, error distribution plots, box and whisker diagrams, heatmaps, and the cumulative frequency plot. A cross plot shows how well data are distributed around the ideal X = Y line. The X = Y line is a line on which the data must be placed if the prediction is performed ideally. An error distribution plot depicts the relative error versus the experimental value of the data. Prediction accuracy decreases as data deviates from the Y = 0 line.

13 $$Relative \; Error=\frac{{x}_{i,exp}-{x}_{i,pred}}{{x}_{i,exp}}\times 100.$$

Box and whisker diagrams are supposed to illustrate how well the model predicts the refractive index value concerning the four quartiles of a specific anionic or cationic family. The heatmaps also show the distribution of certain parameters with respect to the relative error. The cumulative frequency plot shows what portion of data is subject to less than a certain amount of an absolute relative error. Therefore, the x-axis is the absolute relative error.

14 $$Absolute \; Relative \; Error=\left|\frac{{x}_{i,exp}-{x}_{i,pred}}{{x}_{i,exp}}\right|\times 100,$$

where ${x}_{i,exp}$ is the i-th experimental value of the refractive index and ${x}_{i,pred}$ is the i-th predicted value of the refractive index.

## Results and discussion

### Statistical analysis

The results of this study present the effectiveness of machine learning methods in predicting the refractive indices of a wide range of ILs, which was the original purpose of this study. Six well-known machine learning models based on the chemical structures of numerous ILs were employed to achieve the aim of this study. Although all the models performed satisfyingly, the most accurate ML method was CatBoost; followed by XGBoost, LightGBM, Ada-DT, CNN, and Ada-SVM. Table 5 illustrates the statistical results of the training, testing, and overall data for each model used. The results showed a remarkably low error for most of the models. Among them, CatBoost results with an overall R^2^, AAPRE, MAE, and RMSE of 0.9973, 0.0545, 0.0008, and 0.0021, respectively, reveal the extraordinary power of this ML model to predict the refractive index of more than 6000 data points. The least accurate model is Ada-SVM, with the statistical specifications of 0.9227 for R^2^, 0.6618 for AAPRE, 0.0097 for MAE, and 0.0115 for RMSE. Although Ada-SVM is the least accurate model used in the current study, the comparison with other studies shows that it is sensibly acceptable.

**Table 5:** The output statistics of each model.

Statistical parameters	Models					
	XGBoost	LightGBM	CNN	CatBoost	Ada-SVM	Ada-DT
Train (4878 points)						
AAPRE	0.1076	0.1198	0.3915	0.0436	0.6604	0.1976
MAE	0.0016	0.0018	0.0057	0.0006	0.0097	0.0029
RMSE	0.0025	0.0034	0.0076	0.0014	0.0114	0.0038
R^2^	0.9963	0.9932	0.9686	0.9988	0.9225	0.9914
Test (1220 points)						
AAPRE	0.1465	0.1507	0.4047	0.0981	0.6677	0.2347
MAE	0.0022	0.0022	0.0059	0.0014	0.0098	0.0034
RMSE	0.0037	0.0041	0.0080	0.0038	0.0117	0.0048
R^2^	0.9925	0.9906	0.9673	0.9919	0.9234	0.9873
Total (6098 points)						
AAPRE	0.1154	0.1260	0.3941	0.0545	0.6618	0.2050
MAE	0.0017	0.0019	0.0058	0.0008	0.0097	0.0030
RMSE	0.0028	0.0035	0.0077	0.0021	0.0115	0.0040
R^2^	0.9955	0.9927	0.9683	0.9973	0.9227	0.9905

A comprehensive comparison of the results of this study and the ones from the literature is demonstrated in Table 6. According to Table 6, this research considered the most ILs for predicting the refractive index, and the number of input data points was higher than in most studies. Thus, the current study is comparable with the one Baskin et al.^39^ did regarding the extensiveness of the input points. However, concerning the errors, this research showed much better results than Baskin et al.’s study^39^. Unlike previous ML studies, our study includes the wavelength as model input. This inclusion takes another essential factor into account for the refractive index prediction. Additionally, a wide range of temperatures was used in our dataset, giving the study an edge over the survey of Baskin et al.^39^.

**Table 6:** Detailed description of the reported results of the literature and the present study.

References	RMSE	R^2^	AAPRE	MAE	Number of data points	Number of ILs	Model
This research	0.0028	0.9955	0.1154	0.0017	6098	483	XGBoost
This research	0.0035	0.9927	0.1260	0.0019	6098	483	LightGBM
This research	0.0077	0.9683	0.3941	0.0058	6098	483	CNN
This research	0.0021	0.9973	0.0545	0.0008	6098	483	CatBoost
This research	0.0115	0.9227	0.6618	0.0097	6098	483	Ada-SVM
This research	0.0040	0.9905	0.2050	0.0030	6098	483	Ada-DT
Koller et al.^21^	0.007	–	–	–	32	10	Correlation
Safdar et al.^22^	–	more than 0.99	–	–	35	–	Correlation
Tong et al.^23^	–	0.99	–	–	25	1	Semiempirical method
Xu et al.^24^	–	0.99	–	–	35	1	Semiempirical method
Gardas et al.^25^	–	–	0.18	–	245	24	Group contribution method
Almeida et al.^26^	–	–	0.02	–	105	7	Group contribution method
Sattari et al.^27^	9.97 × 10^–3^	0.964	0.34	–	931	97	Group contribution method
Díaz-Rodríguez et al.^28^	–	0.98	0.24	–	72	–	Multilayer perceptrons (MLP)
Díaz-Rodríguez et al.^28^	–	0.76	0.72	–	72	–	Multiple linear regression (MLR)
Díaz-Rodríguez et al.^29^	–	more than 0.99	0.02	–	156	3 (binary mixtures containing ILs)	Multilayer perceptrons (MLP)
Díaz-Rodríguez et al.^30^	–	–	less than 0.48	–	39	39	MLP models
Cancilla et al.^32^	–	–	0.05	–	72	1 (ternary mixtures containing IL)	ANN
Cancilla et al.^33^	–	–	less than 1	–	146	–	Four different models based on MLPs
Soriano et al.^34^	–	–	–	0.00783	752	19 (binary mixtures containing ILs)	ANN
Mesbah et al.^12^	–	0.9225	0.2773	–	362	1 (ternary mixtures containing IL)	Artificial neural networks (ANNs)
Mesbah et al.^12^	–	0.9765	0.1383	–	362	1 (ternary mixtures containing IL)	Gene expression programming (GEP)
Kang et al.^35^	–	0.841	0.855	–	1194	115	Multiple linear regression (MLR)
Kang et al.^35^	–	0.957	0.295	–	1194	115	Extreme learning machine (ELM)
Venkatraman et al.^10^	–	more than 0.85	–	less than 0.01	3147	467	Ensemble decision tree-based machine learning models
Soroush et al.^11^	–	0.9993	0.04	–	812	5 (ternary mixtures containing ILs)	ANN
Wang et al.^36^	–	0.9905	2.42	–	688	5 (binary mixtures containing ILs)	MLP
Sattari et al.^9^	1.07 × 10^−2^	0.935	0.51	–	931	97	QSPR
Wang et al.^37^	–	0.961	0.179	–	2138	299	GC-ANN
Wang et al.^37^	–	0.886	0.628	–	2138	299	GC
Ding et al.^38^	0.017	0.782	–	–	3147	467	Morgan fingerprint-based XGBoost-assisted QSAR
Ding et al.^38^	0.016	0.836	–	–	3147	467	Atom-pair fingerprint-based XGBoost-assisted QSAR
Ding et al.^38^	0.013	0.853	–	–	931	97	Morgan fingerprint-based XGBoost-assisted QSAR
Ding et al.^38^	0.022	0.568	–	–	931	97	Atom-pair fingerprint-based XGBoost-assisted QSAR
Sun et al.^13^	0.0088	0.951	–	–	3964	–	QSPR model developed by molecular fingerprint combined with molecular descriptor (MF_MD)
Sun et al.^13^	0.009	0.948	–	–	3964	–	QSPR model developed by molecular fingerprint (MF)
Sun et al.^13^	0.0108	0.925	–	–	3964	–	QSPR model developed by molecular descriptor (MD)
Sun et al.^13^	0.0107	0.926	–	–	3964	–	QSPR model developed by addition of MF (MF + MF)
Baskin et al.^39^	0.016	0.86	–	0.0081	6443	477	ASNN ML method and CDK23 representation for QSPR model at 25 °C, and melting point
Baskin et al.^39^	0.0112	0.922	–	0.00725	6443	477	DNN ML method and CDK23 representation for QSPR model at 8 different temperatures

**Figure 8:**
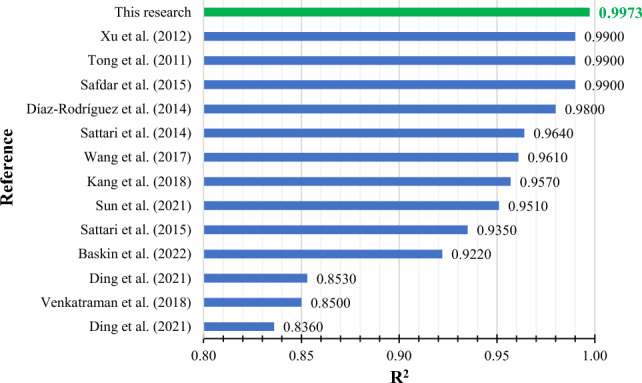
The R^2^ obtained from the CatBoost model of the current study and the literature.

**Figure 9:**
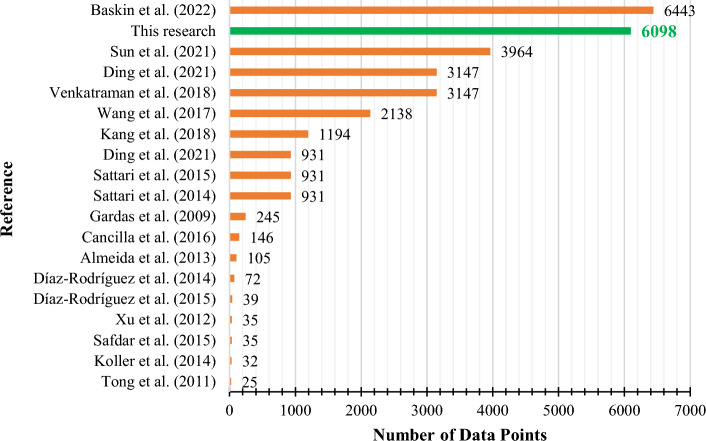
The number of data points used in the current study and the literature.

**Figure 10:**
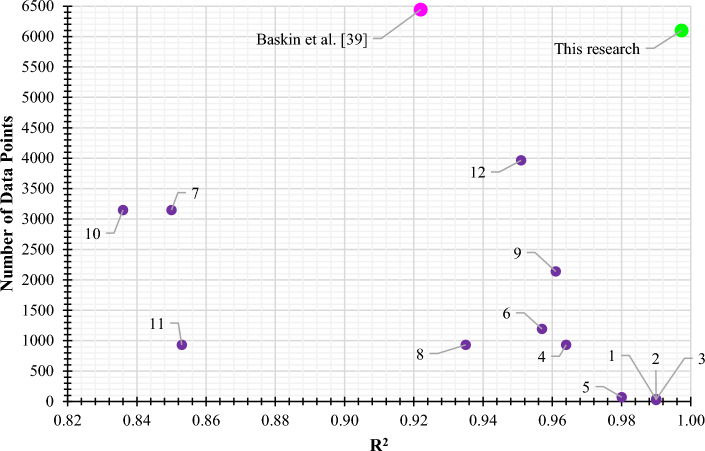
Comparison of the R^2^ and the number of data points between the current study and the literature. The numbers refer to the following: 1: Safdar et al.^22^, 2: Tong et al.^23^, 3: Xu et al.^24^, 4: Sattari et al.^27^, 5: Díaz-Rodríguez et al.^28^, 6: Kang et al.^35^, 7: Venkatraman et al.^10^, 8: Sattari et al.^9^, 9: Wang et al.^37^, 10: Ding et al.^38^, 11: Ding et al.^38^, 12: Sun et al.^13^.

Figures 8 and 9 compare the present study’s R^2^ of the CatBoost model and the number of our data points with the pure ionic liquid studies from the literature, respectively. The superiority of the current research is evident in Figs. 8 and 9 in terms of R^2^ and the number of data points, respectively. Also, Fig. 10 shows these two parameters together in one diagram. The data point number indicates a study’s comprehensiveness, while the R^2^ is a criterion to assess its accuracy. The upper right region of Fig. 10 is where the most accurate and comprehensive studies take place. While Baskin et al.^39^ utilized more data points in their research, their accuracy was lower than the current study’s. It is evident that the best accuracy was obtained in the current study.

In addition to our six main models, we developed an auxiliary MLP model for better comparison with the literature utilizing pure ionic liquids, due to the fact that some studies used MLP models. This research’s MLP model has two hidden layers with 4 and 2 neurons, respectively, and the transfer functions used in the layers are "tansig" for the first hidden layer, "logsig" for the second hidden layer, and "purelin" for the output layer. A comparison between the results of our MLP model and the literature is shown in summarized in Table 7. A closer inspection of the table reveals that the MLP models perform very well with smaller datasets. While it is difficult to fairly compare all the error metrics as the literature did not fully provide their various metrics, our MLP’s results in Table 7 show generally accepted errors. In comparison with other models though, MLP generally might not be as accurate for larger datasets.

**Table 7:** Result comparison of the studies utilized the MLP model and our auxiliary MLP model.

Reference	RMSE	R^2^	AAPRE	MAE	Number of data points	Number of ILs
This research	0.0056	0.9817	0.2450	0.0036	6098	483
Díaz-Rodríguez et al.^28^	–	0.98	0.24	–	72	–
Díaz-Rodríguez et al.^30^	–	–	Less than 0.48	–	39	39
Cancilla et al.^33^	–	–	Less than 1	–	146	–

### Graphical analysis

**Figure 11:**
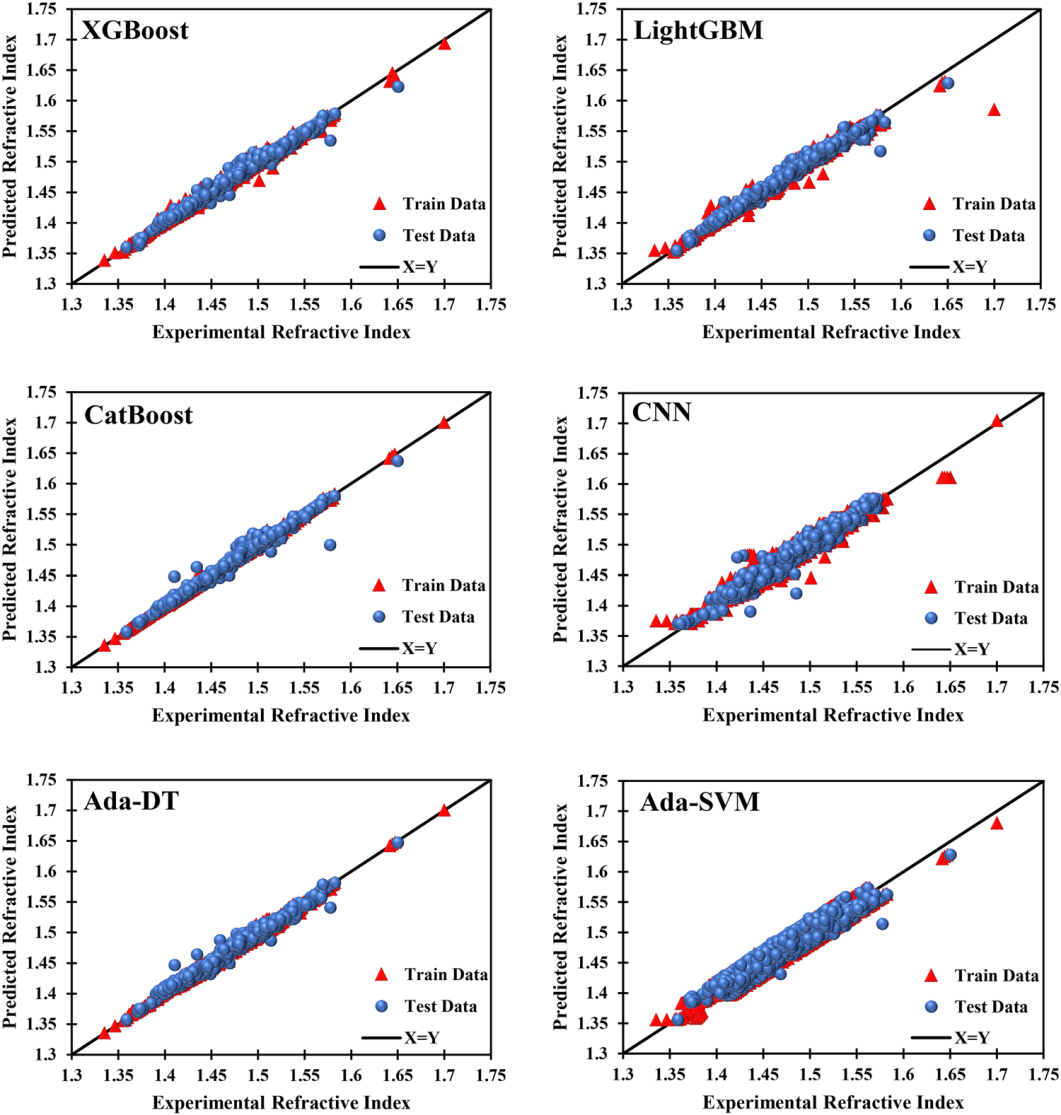
Cross plots of the six models.

**Figure 12:**
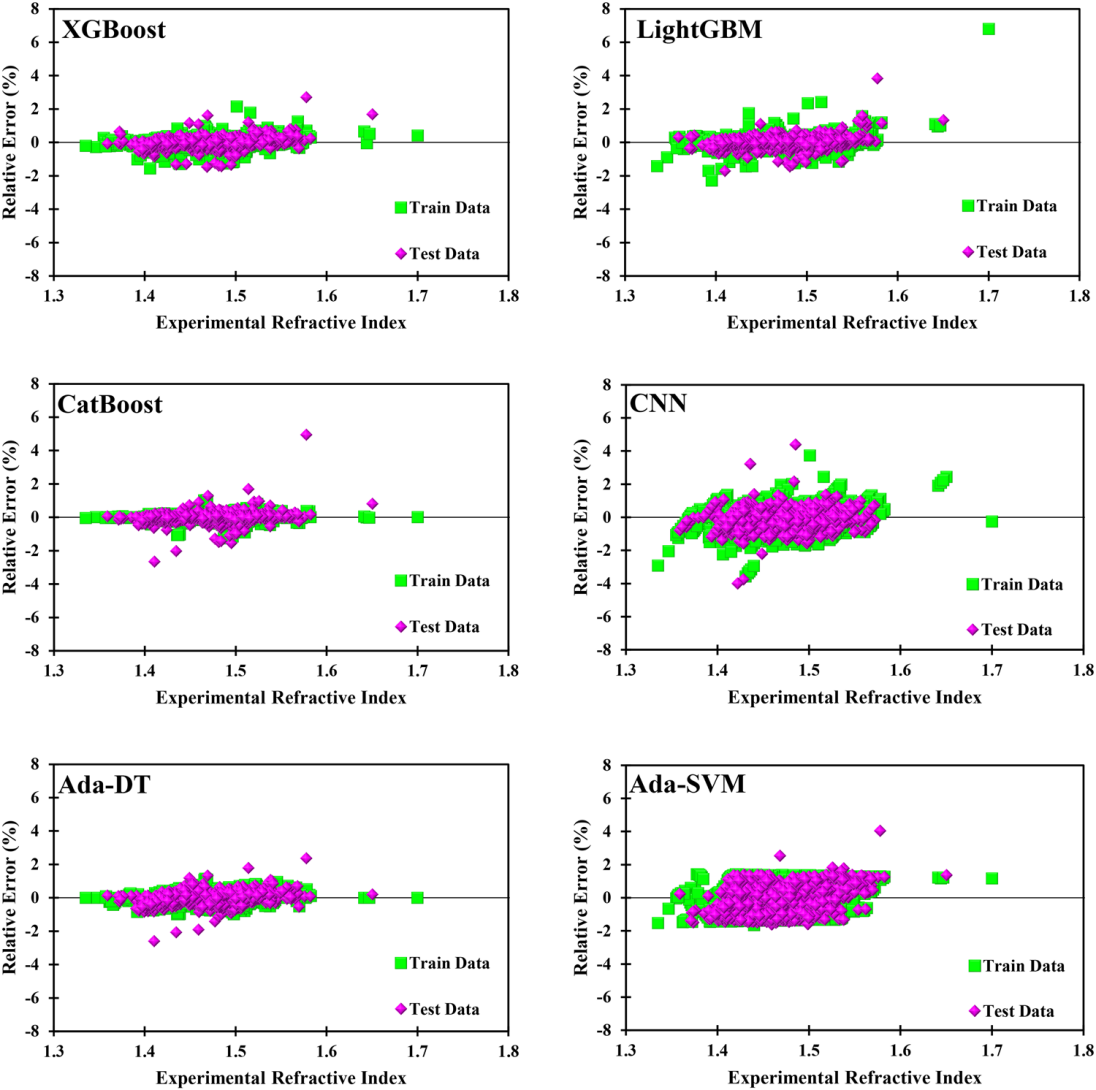
Error distribution plots of the six models.

Graphical analysis is provided to demonstrate the results in another unambiguous way. Figure 11 exhibits the deviance of the data from the ideal X = Y line. The closer the data gets to this line, the better the prediction. A visual inspection of Fig. 11 discloses the accuracy of the used models. Another finding of Fig. 11 is the superior accuracy of the CatBoost model. The error distribution diagrams shown in Fig. 12 lay out the relative error of the predicted refractive index concerning the experimental data. Again, the results are satisfactory since most points have a relative error of less than 2%. A visual comparison confirms that the best model is CatBoost, and the worst is Ada-SVM.

**Figure 13:**
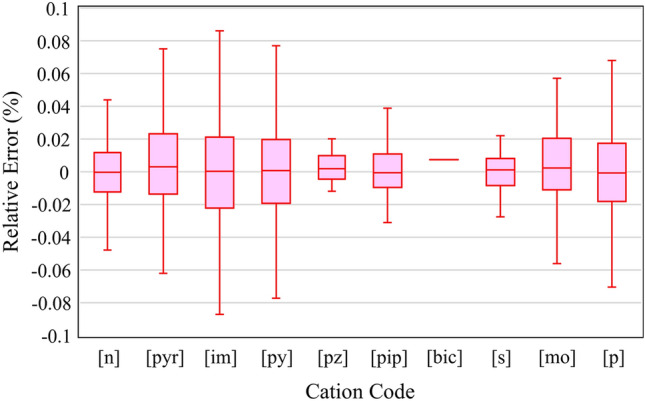
Box and whisker diagram of the relative error against the different cation families. The diagram shows four quartiles without the outlier points.

**Figure 14:**
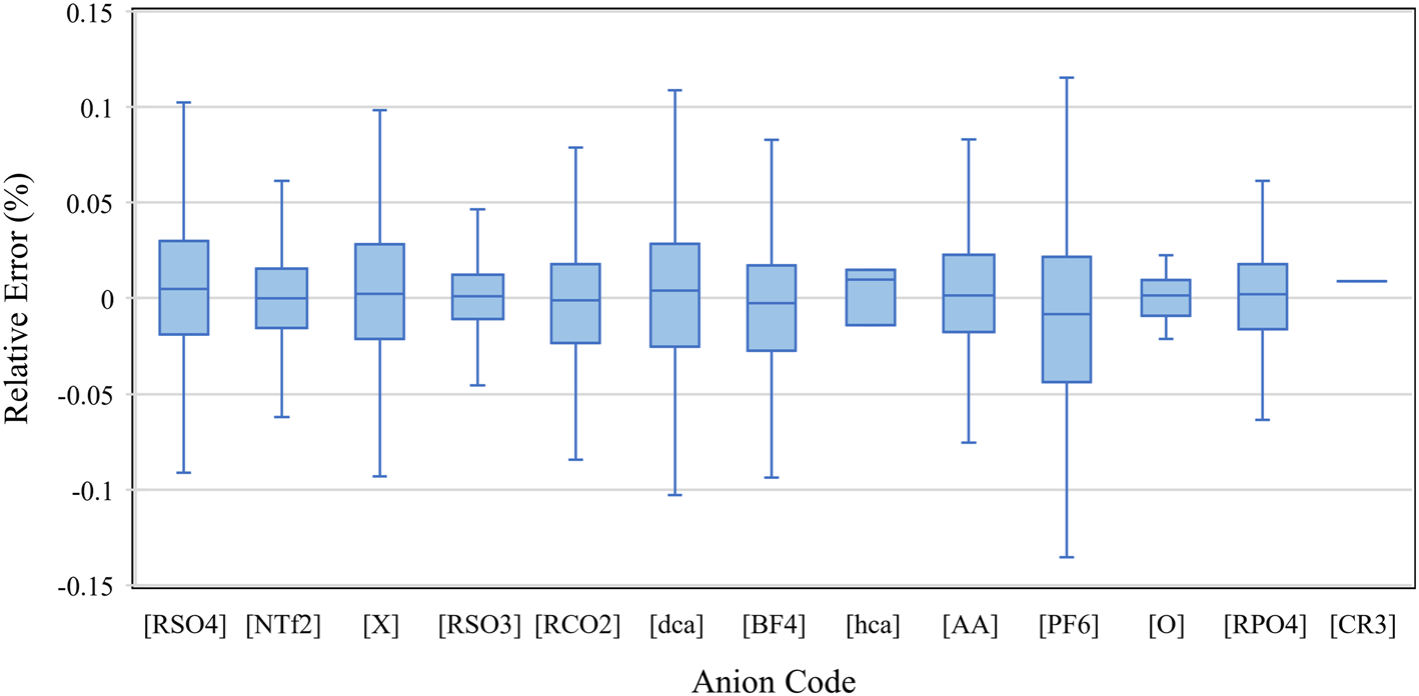
Box and whisker diagram of the relative error against the different anion families. The diagram shows four quartiles without the outlier points.

In addition, the relative errors of the four quartiles of each cation and anion family using CatBoost model are presented in Figs. 13 and 14, respectively. The box and whisker diagrams show that the data in all quartiles of both cation and anion families can predict the refractive index correctly due to their low relative errors. The central marks on the boxes indicate the median. The boxes show the second and third quartiles, and the whiskers and outliers illustrate the first and fourth quartiles of the data. Outlier points are not visible in the figures.

**Figure 15:**
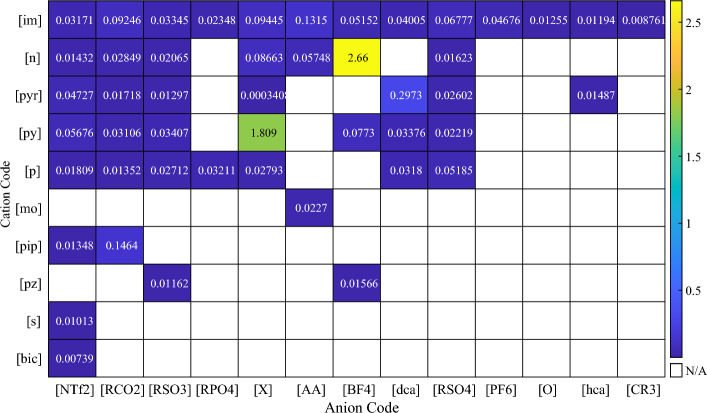
Mean absolute relative errors of every cation–anion family pair.

**Figure 16:**
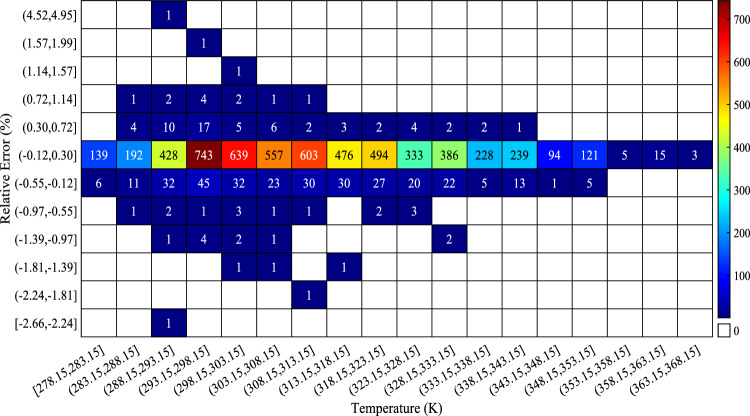
Relative error distribution as a function of temperature.

Figure 15 illustrates the dataset’s mean absolute relative errors for each cation–anion family pair using the CatBoost model. Most ionic liquid families have low mean absolute relative errors. At the same time, a lack of sufficient data points in some specific ionic liquid families resulted in higher mean absolute relative error values, which are still acceptable. The distribution of relative error as a function of temperature utilizing the CatBoost model is shown in Fig. 16. As expected, most data points are located near the relative error equal to zero, such that 743 data points are in the temperature range of 293.15–298.15, with relative errors between -0.12% and 0.30%. Also, the diagram displays that very few data points have large relative errors even though the most significant relative error is less than 5%, which is acceptable.

**Figure 17:**
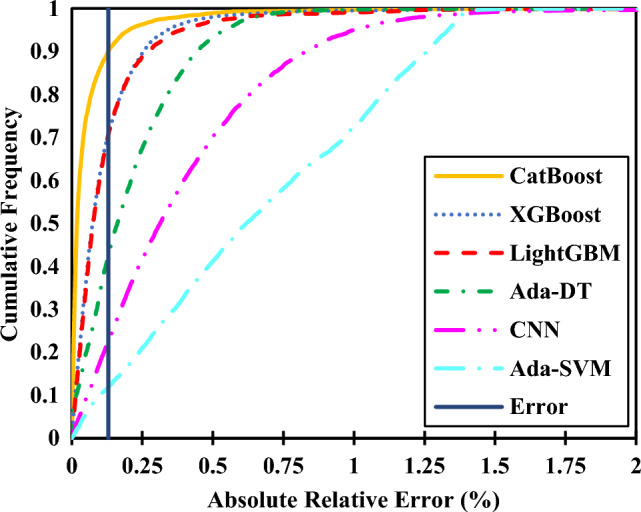
Cumulative frequency plot against absolute relative error.

An additional informative diagram demonstrating a suitable model comparison is shown in Fig. 17. As mentioned before, the CatBoost model performs better than the others regarding the refractive index prediction of ILs. The dashed line of absolute relative error (Error) indicates the value of 0.13%, meaning that 90% of the data analyzed with our best model, CatBoost, have a phenomenal absolute relative error of less than 0.13%. The diagram also illustrates that the most accurate model was CatBoost, followed by XGBoost, LightGBM, Ada-DT, CNN, and Ada-SVM.

### Sensitivity analysis

**Figure 18:**
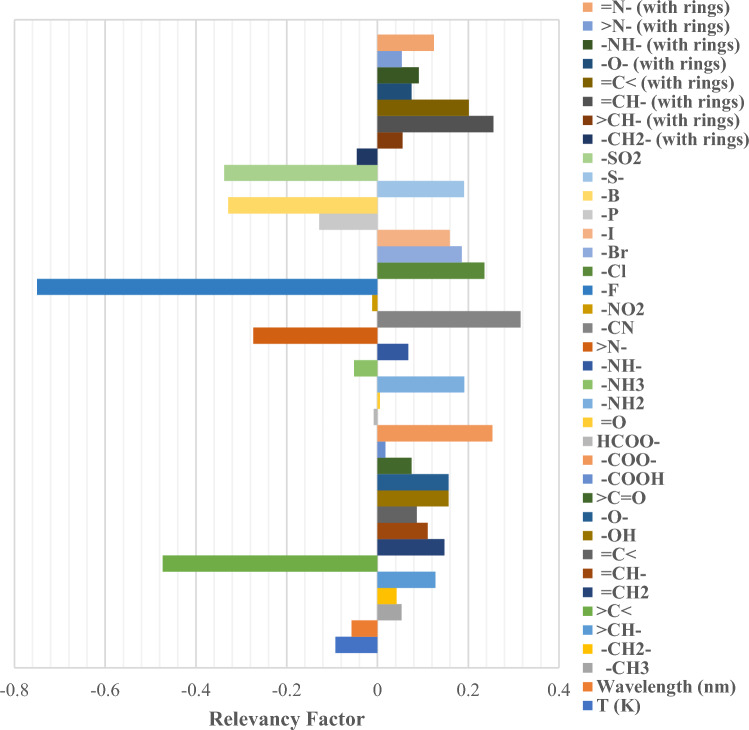
Relevancy factors of the inputs up to down corresponding to their names.

Understanding the significance of each input on the output requires a sensitivity analysis of the results. Here, a relevancy factor was used, which is defined as follows^58^:

15 $$r\left({x}_{v},y\right)=\frac{\sum_{i=1}^{n}\left({x}_{v,i}-\overline{x }\right)\left({y}_{i}-\overline{y }\right)}{\sqrt{\sum_{i=1}^{n}{\left({x}_{v,i}-\overline{x }\right)}^{2}\sum_{i=1}^{n}{\left({y}_{i}-\overline{y }\right)}^{2}}},$$

where ${y}_{i}$ and $\overline{y }$ are the i-th value and the average value of the predicted refractive index, respectively; and ${x}_{v,i}$ and $\overline{x }$ are the i-th value and average value of the v-th input. The input with the highest absolute relevancy factor impacts the output amount most. Figure 18 shows a complete set of the models’ inputs and their corresponding relevancy factors considering the CatBoost model. The colors and the names correspond from top to bottom to avoid confusion. As the figure depicts, the most substantial impact on the refractive index is generated by –F and > C < substructures with a corresponding relevancy factor of − 0.75 and − 0.47, respectively. The notation “with rings” in Fig. 18 implies that the mentioned chemical substructure is located inside a ring in the chemical structure, as defined by Valderrama et al.^56^. In addition, if the r factor of any input is negative, it decreases the refractive index and vice versa. The collected absolute amounts of these factors can be seen in Table 8. Table 8 clearly indicates that temperature and wavelength are not amongst the first half of the most dominant factors influencing the refractive index.

**Table 8:** Input ranking regarding their absolute relevancy factors.

Rank	Input	$\left|\mathrm{r}\right|$	Rank	Input	$\left|\mathrm{r}\right|$
1	–F	0.7499	20	=N– (with rings)	0.1243
2	>C<	0.4732	21	=CH–	0.1104
3	–SO_2_	0.3378	22	T (K)	0.0923
4	–B	0.3289	23	–NH– (with rings)	0.0914
5	–CN	0.3154	24	=C<	0.0868
6	>N–	0.2737	25	–O– (with rings)	0.0750
7	=CH– (with rings)	0.2556	26	>C=O	0.0749
8	–COO–	0.2533	27	–NH–	0.0681
9	–Cl	0.2353	28	Wavelength (nm)	0.0573
10	=C< (with rings)	0.2010	29	>CH– (with rings)	0.0549
11	–NH_2_	0.1912	30	>N– (with rings)	0.0533
12	–S–	0.1909	31	–CH_3_	0.0531
13	–Br	0.1857	32	–NH_3_	0.0515
14	–I	0.1592	33	–CH_2_– (with rings)	0.0455
15	–OH	0.1563	34	–CH_2_–	0.0419
16	–O–	0.1562	35	–COOH	0.0174
17	=CH_2_	0.1476	36	–NO_2_	0.0115
18	–P	0.1285	37	HCOO–	0.0086
19	>CH–	0.1274	38	=O	0.0051

The relevance importance rank of the wavelength is 28th among 38 factors. Although it did not gain a high rank, the importance of the wavelength is not ruled out. The fact that the existence or the quantity of certain substructures outranks the wavelength emphasizes the necessity of choosing appropriate materials, not the negligible role of wavelength. Once the material is chosen (i.e. all the substructures remain constant), the wavelength change shows its significant role.

The Pearson correlation coefficient used here is especially useful when the data distribution is normal. Otherwise, the correlation coefficients are advised to be calculated from the ranks of the data instead of their actual values. The coefficients recommended for this goal are Kendall's tau and Spearman's rho. Since some researchers suggest that Kendall's tau may draw more accurate generalizations compared to Spearman's rho^59^, the absolute Kendall's tau is reported in Table 9. Kendall’s tau formula in case of tied ranks is as follows^60^:

16 $${\tau }_{B}=\frac{2\times \left({N}_{a}-{N}_{d}\right)}{\sqrt{\left[N\left(N-1\right)-\sum {t}_{x}\left({t}_{x}-1\right)\right]\times \left[N\left(N-1\right)-\sum {t}_{y}\left({t}_{y}-1\right)\right]}},$$

where ${\tau }_{B}$ is Kendall's tau with tied rank adjustments, ${N}_{a}$ is the number of agreements in order, ${N}_{d}$ is the number of disagreements in order, $N$ is the number of data points, and ${t}_{x}$ and ${t}_{y}$ are the number of tied observations on the first and second variables, respectively.

**Table 9:** Input ranking regarding their absolute Kendall’s tau.

Rank	Input	$\left|{\uptau }_{\mathrm{B}}\right|$	Rank	Input	$\left|{\uptau }_{\mathrm{B}}\right|$
1	–F	0.6279	20	Wave length (nm)	0.1020
2	>C<	0.3560	21	>C=O	0.0939
3	–CN	0.3125	22	=N– (with rings)	0.0841
4	–B	0.2827	23	–NH– (with rings)	0.0814
5	–SO_2_	0.2424	24	–I	0.0787
6	–COO–	0.2249	25	>CH– (with rings)	0.0780
7	>N–	0.2119	26	–O– (with rings)	0.0733
8	–Cl	0.1955	27	–NH–	0.0691
9	–NH2	0.1762	28	T(K)	0.0670
10	=C< (with rings)	0.1714	29	–CH2–	0.0591
11	–O–	0.1637	30	–CH3	0.0545
12	=CH– (with rings)	0.1606	31	=C<	0.0529
13	>CH–	0.1473	32	–CH2– (with rings)	0.0310
14	–OH	0.1437	33	–NH3	0.0212
15	–Br	0.1430	34	=O (other)	0.0194
16	–P	0.1209	35	–COOH	0.0095
17	–S–	0.1098	36	–NO_2_	0.0047
18	=CH_2_	0.1097	37	>N– (with rings)	0.0038
19	=CH–	0.1051	38	HCOO–	0.0009

While some of the ranks have changed compared to Table 8 due to the correlation method switch, Table 9 consolidates the fact that the –F and > C < substructures are highly correlated with the refractive index.

### Trend analysis

Understanding the influence of changing input values on the output is another illuminative way of analyzing the results. This insight can be gained from the trend analysis of the alkyl chain length, temperature, and wavelength. All parameters except the considered one are fixed to display only the effect of the parameter in question. Additionally, the CatBoost model was used in trend analysis as it is our most accurate model.

**Figure 19:**
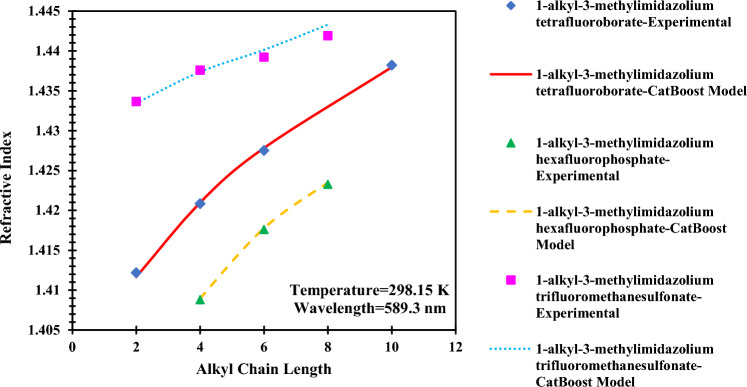
Effect of changing the alkyl chain length of the cation on the refractive index of imidazolium-based ILs.

Figure 19 shows the trend of refractive index with respect to the number of carbons in the cation of three ILs named 1-alkyl-3-methylimidazolium tetrafluoroborate^61^, 1-alkyl-3-methylimidazolium hexafluorophosphate^62^, and 1-alkyl-3-methylimidazolium trifluoromethanesulfonate^63^ for experimental and predicted data. According to Fig. 19, the refractive index rises with the increase in the alkyl chain length of the cation of imidazolium-based ionic liquids. This behavior is due to the molar refraction variation with the number of carbon atoms^1^.

**Figure 20:**
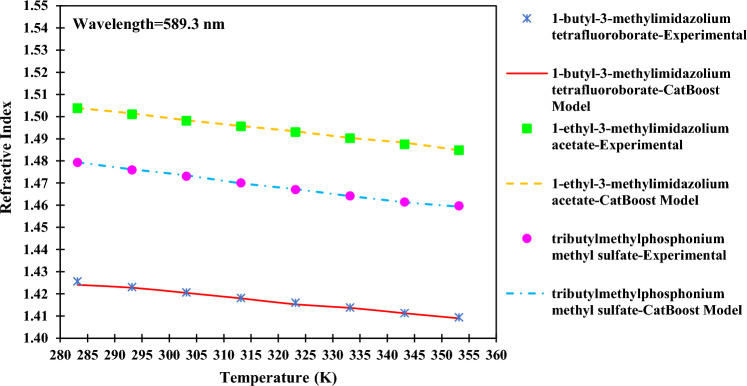
Effect of changing the temperature on the refractive index of ILs.

Demonstrating the effect of temperature on the refractive index was done by considering 1-butyl-3-methylimidazolium tetrafluoroborate^64^, 1-ethyl-3-methylimidazolium acetate^65^, and tributylmethylphosphonium methyl sulfate^66^ in Fig. 20. Unlike the alkyl chain length, an increase in ILs’ temperature reduces the refractive index value. This phenomenon happens because when the temperature rises, the density of the ionic liquid decreases, which consequently increases the free molar volume. This increase in the free molar volume causes the refractive index reduction^1^. This result is in accordance with what the literature concludes^36^.

**Figure 21:**
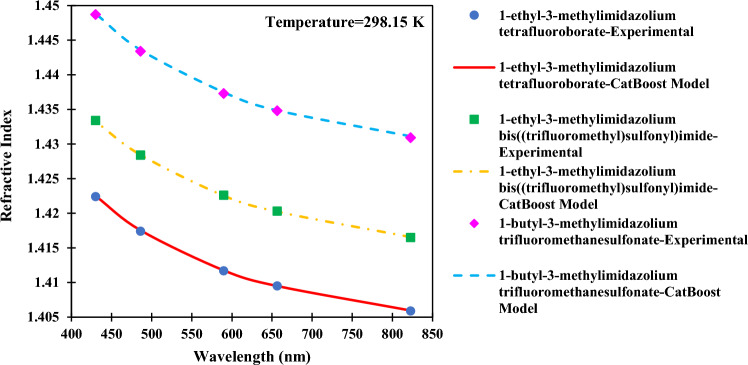
Effect of changing the wavelength on the refractive index of ILs.

Finally, the effect of wavelength change on the refractive index has been illuminated in Fig. 21. Three chosen ILs to display this effect are 1-ethyl-3-methylimidazolium tetrafluoroborate, 1-ethyl-3-methylimidazolium bis((trifluoromethyl)sulfonyl)imide, and 1-butyl-3-methylimidazolium trifluoromethanesulfonate^1^. Observation of refractive index decrease with wavelength increase confirms what the literature states as normal dispersion^67^.

### Leverage approach

Leverage analysis is a method that could reveal outliers and the approximate range within which a prediction is likely to be accurate. Identifying the leverage points is essential because they might influence the prediction considerably. The hat matrix needs to be introduced as follows to determine the leverages of the inputs^68^:

17 $$H=X{\left({X}^{T}X\right)}^{-1}{X}^{T}.$$

The diagonal elements of the hat matrix are named leverages and satisfy:

18 $$0\le {h}_{ii}\le 1,$$

where ${h}_{ii}$ is the diagonal elements of the hat matrix. A threshold can be introduced as the upper limit of the standard values, which is usually defined as:

19 $${H}^{*}=\frac{3(a+1)}{n},$$

where $a$ and is the number of inputs and $n$ is the number of data points^69^. The following equation calculates standardized residuals:

20 $${R}_{i}=\frac{{e}_{i}}{\sqrt{MSE\left(1-{h}_{ii}\right)}},$$

where $MSE$ is the mean square error and ${e}_{i}$ is the ordinary residual of the i-th observation. As an accepted standard used by researchers, if the absolute value of a data point’s standardized residual is less than 3, the data is considered valid. The data out of this boundary is considered to be suspected^70^.

**Figure 22:**
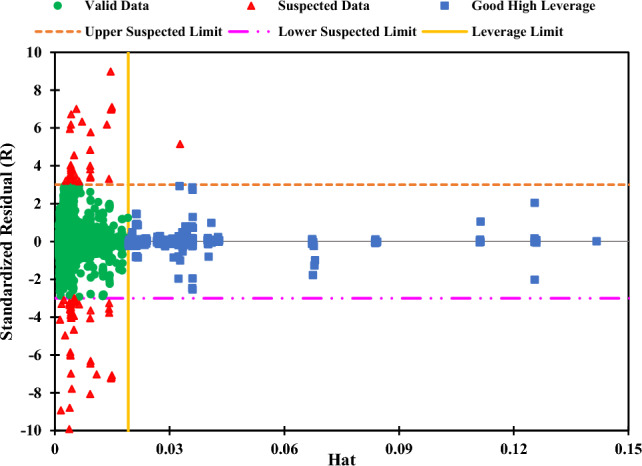
Williams plot of the CatBoost model’s results.

The Williams diagram can be plotted in Fig. 22 thanks to the available leverages and standardized residuals. The lines R = 3 and R = − 3 are drawn to indicate the limits of the valid data. Also, to emphasize the limit beyond which the data points have high leverages, Hat = 0.019 is displayed. With these criteria, 95 out of 6098 data points (roughly 1.5%) were detected as suspected data, and 273 were good high leverage points (approximately 4.5%). So the number of valid data was 5730 (about 94% of the total data). This finding shows that a small portion of the data was not reasonable, and the CatBoost model’s performance was impressive. Setting the y-axis and x-axis to display data in the range of [− 10, 10] and [0, 0.15], respectively, can help the data points variation to be displayed clearly. Four points have an R of less than − 10, two points have an R of more than 10, and one point has a leverage value of more than 0.15. These seven points are not shown in Fig. 22, but the supplementary data section provides the entire plot (Fig. S1). The dataset has an unusual point with a very high Hat value (around 0.33). The point belongs to an ionic liquid with three -I substructures, unprecedented in the dataset. Because the leverage method only focuses on the inputs regardless of the output amounts, this anomaly in the inputs escalates the leverage value.

## Conclusions

This study aimed to predict the refractive index of an abundant number of ILs. As a novel approach, the wavelength and 36 chemical substructures were considered as inputs, along with the temperature. More than 6000 data points were gathered and used in 6 different chemical structure-based machine learning models named XGBoost, LightGBM, CatBoost, CNN, Ada-DT, and Ada-SVM to achieve this study’s aim. The results’ statistical and visual analysis reveals that the most accurate model was CatBoost. Other models also performed effectively and can be sorted as XGBoost, LightGBM, Ada-DT, CNN, and Ada-SVM regarding their accuracy. Other findings of the research are highlighted as follows:

- The sensitivity analysis showed that the –F and > C < substructures have the most influence over the predicted refractive index of an ionic liquid. The presence of these substructures in an ionic liquid declines the amount of the refractive index.
- Apart from the type of ILs, the temperature has a more powerful effect in calculating the refractive index than the wavelength.
- Neither temperature nor wavelength was among 50% of the most influential inputs on the refractive index. The type of ionic liquid, most precisely, the presence of certain chemical substructures, had more impact on the output than temperature and wavelength.
- The results of the leverage approach display that some points have uncommon leverage values. This occurrence could result from abnormal chemical substructures in the ILs.
- Using machine learning methods for predicting the refractive index of a vast number of ILs showed an extraordinary performance that even our worst model’s performance was acceptable with an R^2^ of 0.9227 and an AAPRE of 0.6618. At the same time, our best model’s statistical results were exceptional, with an R^2^ of 0.9973 and an AAPRE of 0.0545.
- The trend analysis reveals that the refractive indices of ILs decline with wavelength and temperature rise while the refractive indices of imidazolium-based ILs increase with the alkyl chain length increase.

## Supplementary Information

Supplementary Information 1.

Supplementary Information 2.

Supplementary Information 3.

Supplementary Information 4.

Supplementary Information 5.

Supplementary Information 6.

Supplementary Information 7.

Supplementary Information 8.

Supplementary Information 9.

Supplementary Information 10.

## Data Availability

All data generated or analyzed during this study are included in this published article (and its Supplementary Information files).
