# Supplementary material for: Insights into modeling refractive index of ionic liquids using chemical structure-based machine learning methods
Source: Sci Rep. 2023 Jul 24;13:11966. doi: 10.1038/s41598-023-39079-5 (PMC10366230; doi:10.1038/s41598-023-39079-5)
Supplement: Supplementary file 3 — Supplementary Information 3. [file 41598_2023_39079_MOESM3_ESM.docx]

**Appendix**

In this section, the implementation of our best model (CatBoost) is described. First, the “catboost” library needs to be installed and imported in Python, as well as “pandas” and “sklearn”. The “pandas” library helps to manipulate the data while “sklearn” library is used for splitting the dataset to train and test parts. Then, training the model by the obtained optimized values and predicting the target value is performed using the “catboost” library. And finally, an example of the method to test the model with a candidate data point is provided. The Python code can be found in the Supplementary Information files.
