# Supplementary material for: Insights into modeling refractive index of ionic liquids using chemical structure-based machine learning methods
Source: Sci Rep. 2023 Jul 24;13:11966. doi: 10.1038/s41598-023-39079-5 (PMC10366230; doi:10.1038/s41598-023-39079-5)
Supplement: Supplementary file 9 — Supplementary Information 9. [file 41598_2023_39079_MOESM9_ESM.docx]

**Insights into Modeling Refractive Index of Ionic Liquids using Chemical Structure-based Machine Learning Methods**

Ali Esmaeili ^a,1^, Hesamedin Hekmatmehr ^a,1^, Saeid Atashrouz ^b,*^, Seyed Ali Madani ^c^, Maryam Pourmahdi ^d^
Dragutin Nedeljkovic ^e^, Abdolhossein Hemmati-Sarapardeh ^f,g,*^, Ahmad Mohaddespour ^h,*^

*^a^ Renewable Energies Engineering Department, Faculty of Mechanical and Energy Engineering, Shahid Beheshti University, Tehran, Iran
^b^ Department of Chemical Engineering, Amirkabir University of Technology (Tehran Polytechnic), Tehran, Iran
^c^ Department of Chemical and Petroleum Engineering, University of Calgary, 2500 University Drive NW, Calgary,
AB T2N 1N4, Canada*

*^d^ Department of Polymer Reaction Engineering, Faculty of Chemical Engineering, Tarbiat Modares University, Tehran, Iran
^e^ College of Engineering and Technology, American University of the Middle East, Kuwait
^f^ Department of Petroleum Engineering, Shahid Bahonar University of Kerman, Kerman, Iran
^g^ State Key Laboratory of Continental Shale Hydrocarbon Accumulation and Efficient Development, Ministry of
Education, Northeast Petroleum University, Daqing 163318, China
^h^ Department of Chemical Engineering, McGill University, Montreal, QC H3A 0C5, Canada*

^1^ Hesamedin Hekmatmehr and Ali Esmaeili contributed equally to this work as first authors.

* **Corresponding authors:** S. Atashrouz (s.atashrouz@gmail.com and saeid_atashrouz@aut.ac.ir), A. Hemmati-Sarapardeh

(hemmati@uk.ac.ir and aut.hemmati@gmail.com), A. Mohaddespour (ahmad.mohaddespour@mail.mcgill.ca)

Figure S1- Full Williams plot of the leverage analysis
